# Untangling the Tauopathy for Alzheimer’s disease and parkinsonism

**DOI:** 10.1186/s12929-018-0457-x

**Published:** 2018-07-10

**Authors:** Hui-Yun Chang, Tzu-Kang Sang, Ann-Shyn Chiang

**Affiliations:** 1 Department of Medical Science, Institute of Systems Neuroscience, 101, Section 2, Kuang-Fu Road, Hsinchu, 30013 Taiwan; 2 Department of Life Science, Institute of Biotechnology, 101, Section 2, Kuang-Fu Road, Hsinchu, 30013 Taiwan; 3 Brain Research Center, National Tsing Hua University, 101, Section 2, Kuang-Fu Road, Hsinchu, 30013 Taiwan

**Keywords:** Microtubule associated protein tau, Brain connectome, Tauopathy, Alzheimer’s disease, Parkinson’s disease, Proteinopahty, trans-synaptic spreading, Mitochondria, age-dependent neurodegenerative diseases

## Abstract

Tau is a microtubule-associated protein that mainly localizes to the axon to stabilize axonal microtubule structure and neuronal connectivity. Tau pathology is one of the most common proteinopathies that associates with age-dependent neurodegenerative diseases including Alzheimer’s disease (AD), and various Parkinsonism. Tau protein undergoes a plethora of intra-molecular modifications and some altered forms promote the production of toxic oligomeric tau and paired helical filaments, and through which further assemble into neurofibrillary tangles, also known as tauopathy. In this review, we will discuss the recent advances of the tauopathy research, primarily focusing on its association with the early axonal manifestation of axonal transport defect, axonal mitochondrial stress, autophagic vesicle accumulation and the proceeding of axon destruction, and the pathogenic Tau spreading across the synapse. Two alternative strategies either by targeting tau protein itself or by improving the age-related physiological decline are currently racing to find the hopeful treatment for tauopathy. Undoubtedly, more studies are needed to combat this devastating condition that has already affected millions of people in our aging population.

## Background

One may or may not develop tauopathy lesion even when his/her family members have Alzheimer’s disease or Parkinsonism– sounds like good news because most tauopathy cases are sporadic [1]. However, it is also a sentiment showing our lack of understanding of this commonly observed proteinopathy in aging brains. The first person who described tauopathy case was the legendary German psychiatrist Alois Alzheimer, he saw his 51-year old patient Auguste, who was disturbed by dementia symptoms which include loss of short-term memory, depression, and delusion. Alzheimer found Auguste’s postmortem brain contained neurofibrillary tangles and senile plaques [2], these proteinopathies later coined as the hallmark of AD pathology. Today we know that Tau proteins make this fibril-like structure. In fact, various forms of Tau aggregation account for more than 20 neurological disorders include AD, frontotemporal dementia with parkinsonism-17 (FTDP-17), progressive supranuclear palsy, corticobasal degeneration, argyrophilic grain disease, chronic traumatic encephalopathy and Parkinson’s disease (PD) [3, 4]. What is the etiology of this devastating pathology formed and how does it affect our brain function? In this review, we summarize the recent advances in tauopathy research and highlight some pathophysiological mechanisms that obviously could cause the damage to our brain in the aging process. The subject selection is biased because of our research data incline specific issues, and we apologize to colleagues for their studies not included [5–7].

**Fig. 1:**
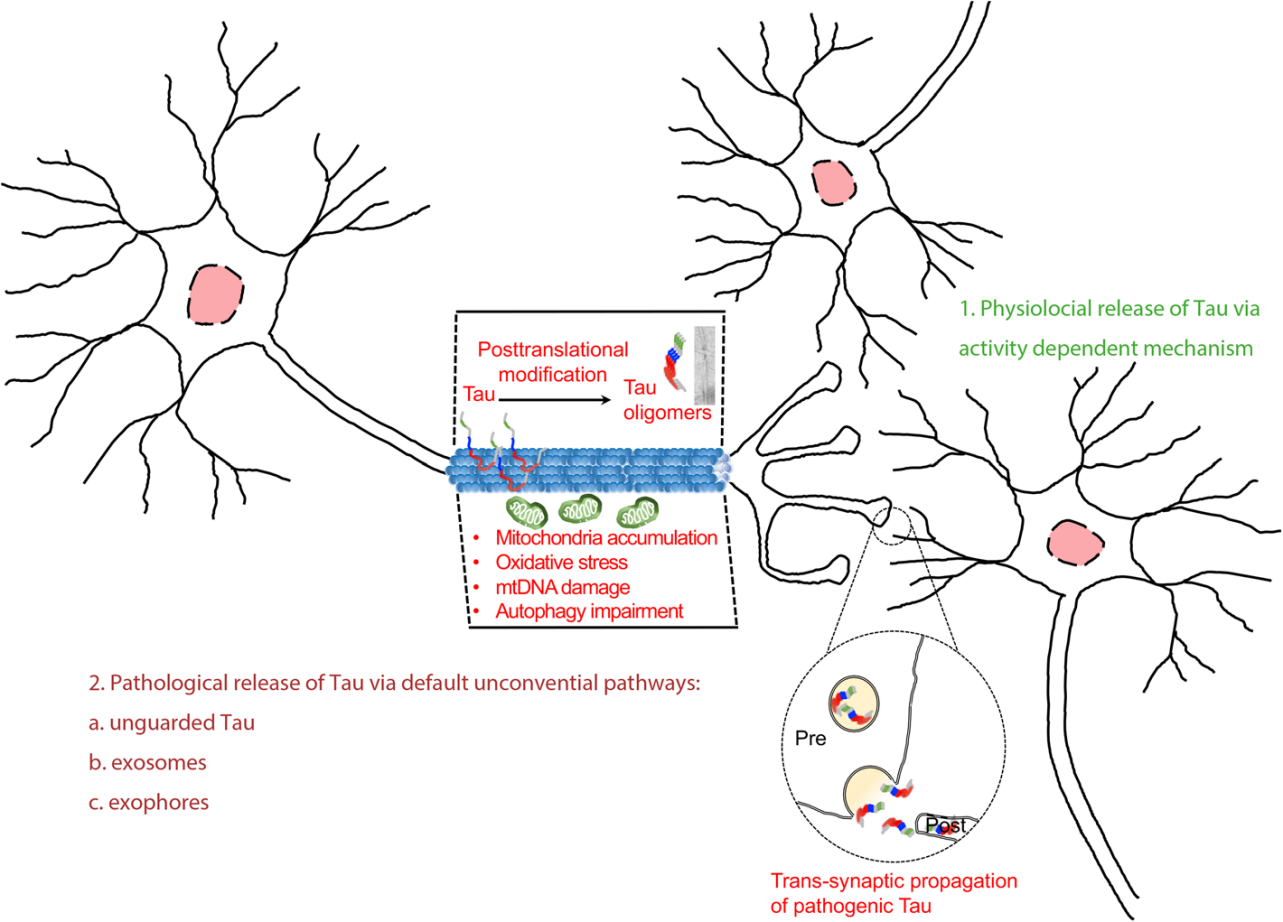
A schematic drawing summarizes possible pathogenic mechanisms in early tauopathy and its subsequent spreading. Tau protein may encounter different post-translational modifications, and some changes could lead to the formation of Tau oligomers and subsequent development of the high-order structure such as pair-helical filament shown in the EM picture. The altered Tau function may cause aberrant mitochondria accumulation, oxidative stress, mtDNA damage, and autophagy impairment in the axon. These conditions damage axonal homeostasis and may grant a route for pathogenic Tau to propagate to the post-synaptic cell. We have incorporated the current knowledge of tau spreading and its potential links to secretory mechanisms. In physiological condition, Tau secretion is activity dependent (23). We have incorporated the current knowledge of tau spreading and its potential links of related secretory mechanisms: unguarded Tau (66); exosome (108); exophore (109)

## Neuronal connectivity

The emergence of an animal brain is the ingenuity of numerous neurons wiring together to form networks that relay and process external and internal stimuli. Our brain comprises an estimated 10~ 100 billions of neurons, and each neuron has hundreds to thousands of synapses that connect to others. In a complex, macro-scaled neuronal network of cortico-cortical pathways, Drs. Olaf Sporns and Patric Hagmann proposed the term “connectome” to conceptualize the comprehensive map of structural connections emerging from the ideas that can be traced back to neuronal connectionism in the early twentieth Century, including studies by Santiago Ramón y Cajal on the microscopic structure of brains [8, 9]. The human brain has an estimated more than a hundred trillion synapses that can be represented as 10^14^ nodes. In a micro-scaled connectome, cells in the network undergo amazing morphological changes during neuronal cell differentiation by forming thousands of synapse and multiple dendrites, and these structures are deemed to be dynamic throughout the lifetime [10, 11]. Any mature neuron is believed to have a stable common architecture, yet it also exhibits a unique individual feature upon the changing of circumstances which often refers as “functional connectome” [12]. It is this complexity that controls our motor, mental, and cognitive functions. Therefore, the maintenance of axonal connectivity, synapse structures, and proper gene expression is critical to the function of a brain. Although an axon and its synapses carry out an unchallenged role as communication outputs, how a neuron determines its unique gene expression, connectivity and the size and strength of synapses remain the fundamental questions need to be solved in neurosciences [13, 14].

Here we review what is known about the potential “trans-synaptic Tau spreading hypothesis” in many devastating neurological conditions, especially in age-related disorders such as AD and PD, of both are prevalent with connectomic pathologies [15] thus reflect the importance of trans-synaptic organization in maintaining brain function. The seminal publications by Heiko Braak and Eva Braak described Tau pathology may initially cause neuritic damage in the entorhinal cortex (EC), from where it spreads to broader brain regions of the hippocampus, and progressively to inferior frontal and parietal cortex [16, 17]. Modern macro-connectomic analysis supported that Tauopathy spreading is preferentially initiated from the densely-connected “hub” regions in the brain, therefore implicating a likelihood of disrupting the evolutionary conserved motor and cognition controls [18]. The EC and the hippocampus are intimately connected brain areas which ensemble properties of grid cells and place cells that give rise to the functional circuitry of map-like spatial environment that an individual can use that for spatial navigation [19, 20]. Tau pathology spreads from the EC could cause deficits in grid cell firing and disrupt the spatial cognition in mice, similar to what being seen in AD [21]. Indeed, it is thought that pathological Tauopathy accumulation in the EC is the first synapse where tau seeds influence memory circuits in the brain, and such pathological evolvement is associated with a progressive loss of episodic memory in AD (Fig. 1) [22]. From the view of tau structure-function relationship in the neuronal circuits, it seems worth to point out that Tau has been shown to involve in the activity-dependent changes of pre-synaptic neuronal communication via its releasing (Fig. 1) [23, 24]. The underlying mechanism that coupling neuronal activity and tau release may associate with the classical synaptic vesicular transmission. However, whether this released tau enters postsynaptic neurons and how does it impact on synaptic plasticity, or maybe to other structural and functional aspects remain to be elucidated.

## Microtubule-associated protein tau

Tau was firstly discovered via co-purification of proteins that associate with microtubule from the brain lysate, by Mark Kirschner and colleagues [25, 26]. Human Tau has six isoforms in the brain with either three or four microtubule-binding repeats (3R or 4R) at the C- terminal. Different Tau isoforms may or may not contain either one or two 29-residue fragments (0 N, 1 N, and 2 N) at the N-terminus, which follow by the proline-rich domain. In fetus brains, only the smallest isoform Tau0N3R can be detected, while all Tau isoforms (0 N3/4R; 1 N3/4R; 2 N3/4R) are reported in the adult brains [27]. Under physiological condition, Tau exists in an unfolded state, and 80% of Tau proteins interact with microtubules in the axons [28]. When Tau is not interacting with other proteins, it may wrap on its own, and this random intra-molecular binding state is believed to be necessary for preventing interactions with additional Tau protein by masking the microtubule-binding repeats. The protein itself is bipolar; the N-terminal domain is highly negative-charged in an isoelectric point of 3.8, whereas the proline-rich domain and microtubule-binding repeats are positive-charged in an isoelectric charge point of 11.4 and 10.8, respectively [29]. It appears that post-translational modifications could modify the charge of Tau and its binding state [30]. The phosphorylation of some residues in the microtubule-binding domain may neutralize the positive charge and dissociate tau from microtubules [31, 32]. Tau is very hydrophilic, containing only a small portion of hydrophobic residues. Also, normal Tau protein exhibits no secondary structure, if any is only transient, and the binding interaction of positive-charged Tau and negative-charged residues in microtubule or tubulin dimers could form a helical structure in the microtubule-binding domain of Tau [33].

The role of Tau has extended from stabilizing axonal microtubule to organizing actin microfilament in the synapses [34, 35]. The N and C termini of Tau are projected outward of microtubule when they are associated [36]. The projection domain can associate with the cell membrane and other organelles such as mitochondria and can cross inter-linked microtubule to actin microfilament [37]. Compared to other MAP protein such as MAP2, the shorter projection domain of Tau determines a tighter inter-microtubule spacing of 20–30 nm in axons, whereas the more extended projection domain of MAP2 provides 60–80 nm space in dendrites microtubules [38]. Besides interacting with the cytoskeleton, Tau has been shown to associate with cell membrane complex, bind Src-homology 3 (domain protein such like Src kinase Fyn in dendrites [39], modulate lipid signaling [40], and protect DNA in the nucleus [41, 42].

Several Tau loss-of-function studies in animal models have provided some insights on its role in neurons. The knock-out of tau in mice could lead to defective axonal transport of amyloid precursor protein (APP), intraneuronal iron accumulation, and neuronal loss in the substantia nigra [43]. Moreover, brains lacking the Tau protein could enhance abnormal axonal spheroids in the white matter tracts in a mutated human APP mouse model for AD [44]. It has also been suggested that the precise temporal and spatial regulation of Tau-binding partners enable neurons to control Tau functions, which is critical for neuron function and plasticity [36].

## Tau mutations in human neurological diseases

Tau is one of the most common proteins involved in different neurodegenerative diseases, and nowadays we use tauopathy to call neurological conditions with the pathogenic involvement of tau aggregates [3]. It is estimated that more than 30 million people are currently living with the burden of tauopathy-associated neurological diseases, including AD, PD, FTDP-17, Parkinsonism-dementia complex of Guam and corticobasal degeneration, progressive supranuclear palsy, corticobasal syndrome, and chronic traumatic encephalopathy [45]. Tau gene mutations have been found in the familiar form of frontal temporal dementia (FTD), a generic disorder covers a range of clinical conditions like Pick’s disease, corticobasal dementia, and progressive supranuclear palsy [46–48]. Tau Mutations, which include G272 V, P301L, V337 M, and R406W, were first discovered in the late 1990s in inherited FTDP-17 families [49, 50]. More than a hundred Tau mutations have been identified up till now, and many of pathogenic mutations are localized to the microtubule binding domains. Despite some Genome-wide Association Studies suggested MAPT as a genetic risk factor in AD, most patients carry wild-type Tau, and the link between Tau and AD is mostly a pathological account. How can the naturally unfolded tau proteins aggregate into highly ordered periodic paired helical and straight tau filaments? The high-resolution atomic analyses have recently refined the filaments cores structure. The current model based on the Cryo-EM structure of tau filaments shows that two twin protofilaments comprising about 70 amino acids (residues 306–378 of which corresponding to R3 and R4 of tau proteins) could form a cross beta/beta helix structure [51]. Because this R3 and R4 domain of tau is required for tau to stabilize microtubules, the filamentous tau has lost its physiological function to assemble and stabilize microtubules. The motif VQIVYK (306–311) in R3 is sufficient to “zipper” similar hexapeptides in R4 to form hairpin of two interdigitated beta sheets [52]. Therefore, the genetic mutations that unmask the hexapeptides VQIVYK (306–311) in R3, such as P301L in FTDP-17, may facilitate the aggregate formation to stack beta sheet core [53].

## Post-translational modifications of tau

Patients who suffer from tauopathies rarely bearing mutated *MAPT* gene. Thus, how the normal Tau proteins become dysfunctional and self-assemble into Tau pathology is a mystery. These modifications of Tau are believed to be the culprit of Tau pathogenesis and have been the central area of Tau research for years [54]. The post-translational modifications of Tau could render the protein to lose its native unfolded structure, and by which promote the beta-sheet C form formation to trigger the Tau aggregation [55]. In this process, the modifications changed the interactions between Tau and other proteins and lost its affinity to tubulin, consequently altered axon trafficking and functions [56]. To date, ten types of Tau protein modification have been reported, the commonly detected modifications such as phosphorylation/dephosphorylation, proteolytic cleavage, and acetylation/deacetylation, are the many facets of tau [57, 58]. Furthermore, Tau could be modified by cross-linking through isomerization, ubiquitylation, sulfenylation, glycosylation, nitration, and sumoylation, reflecting its complex post-translational regulations [55]. The following are the most discussed three post-translational modifications:

- *Phosphorylation/dephosphorylation*
  Phosphorylation of Tau protein is always a complicated issue in physiological and pathological states. Phosphorylation and dephosphorylation are the dominant modifications of physiological Tau and may alter its association with microtubules and likely other mechanisms. The 2N4R Tau composes 85 putative phosphorylation sites (45 serines, 35 threonines, and 5 tyrosines), in which 17 Thr-Pro (T/P) or Ser-Pro (S/P) motifs are abnormally hyperphosphorylated in the AD and other tauopathy conditions [59]. These motifs can be phosphorylated by proline-directed protein kinases such as glycogen synthase kinase 3 (GSK3) and cyclin-dependent kinase (CDK). Another microtubule affinity-regulating kinases and Ca^2+^/ calmodulin-dependent protein kinase II could modify the provisional phosphorylation sites near or in the microtubule-binding motif. These modifications often detach Tau protein from microtubules by which may cause toxicity. Phosphorylation of tau by a subtype of tyrosine protein kinase, including Src family kinases such as FYN [60]. FYN-mediated Tau phosphorylation and the binding between Tau and FYN may change its trafficking or binding partners, thereby missorting tau to synapses and somatodendritic compartments and by which to cause synaptic dysfunction [61]. However, the physiological regulation of tau hyperphosphorylation is not only in the disease state as it also occurs naturally during brain development, in the mitotic cell cycle, and at the state of hibernation [62]. Moreover, tau phosphorylations are intertwined with other post-translational modifications such as acetylation, methylation, ubiquitination, sumoylation, and peptidase/protease cleavage, to regulate the protein degradation and aggregation in a somewhat dynamic manner [63].
  Like many kinase-phosphatase substrates, Tau can be phosphorylated by kinases, and their phosphorylation can be reversed by dephosphorylation via phosphatases. Tau protein phosphatases have been identifed like PP2A, PP1, PP2B, PP2C, and PP5 [64]. Notably, these phosphatases are more sensitive to the temperature changes than kinases because enzyme activities of phosphatase decreased exponentially, whereas kinases linearly reduced in hypothermia [65]. Therefore, it should not be a surprise that the microenvironment may affect Tau modifications and Tau spreading [66]. Hyperphosphorylated Tau could due to the decrease of ~ 20–40% PP2A activity [65], which shows the intertwine between kinases and phosphatases controls Tau property. While the mainstream views consider that Tau hyperphosphorylation is toxic to the affected neuron because the nature of AD brain pathology; however, reversing Tau hyperphosphorylation per se may not be sufficient to cure neurodegeneration for the reason that kinase inhibition was unable to block Tau-induced lesion.
- *Truncation*
  Tau protein could be cleaved by caspases at multiple sites including two highly conserved aspartate residues at 314 and 421 (Asp314 and Asp421) [67]. The truncation of tau has distinct effects on tau protein seeding, aggregation, and spreading. It has been shown that 2N4R Tau lacks residues 275–280 (in R3) and 306–311 (in R4) could prevent Tau seeding [68]. In theory, any cleavage of Tau occurs between R3 and R4 could potentially change the progression of tauopathy. It has been reported that FTDP-17-associated mutant Tau is more resistant to aminopeptidases than normal Tau [69]. However, some Tau truncations might prevent its unfolded nature and thereby promote Tau aggregation [70].
- *Acetylation/deacetylation*
  The other layer of Tau protein modification is acetylation and deacetylation [71]. Tau has 23 lysine residues, and 13 lysine sites are in the microtubule-binding domain which includes KXGS preserved in all four repeats. Enzymes like p300 acetyltransferase and NAD^+^-dependent sirtuin of which functions as deacetylase could act on Tau [72]. One pioneer study implied that the lack of Tau acetylation can be associated with argyrophilic grain disease (AGD), a condition of mild dementia [73]. Because AGD brains showed reduced Tau pathology, raising the possibility that the prevention of Tau acetylation in our brains might be a critical defense mechanism against the spreading of tau aggregates [74]. Paradoxically, it has been reported that the abovementioned acetylation motif KXGS is highly ubiquitinated in human AD brain [75], indicating tau acetylation in the microtubule-binding domain could prevent its degradation. Thus, the ubiquitination and acetylation of the same lysine residues in tau may create a complex state for Tau turnover.

## The intimate connection between tau and mitochondrion

The mammalian brain needs much more energy compared to the rest of the body. When the supply is low, balancing energy expenditure in locals creates a challenge for neurons [76]. Thus, reducing neuron connectivity and brain activity might present an adaptation to energy shortfall [77]. Indeed, when animals are in hibernation, neurons in the hippocampus minimize mitochondrial metabolism, and their dendritic fields with less complexity by reducing neuronal connectivity with loss of ~ 50% synapses [78]. ‘A study of European ground squirrels focused on the effects of memory retention after hibernation revealed such physiological adaptation is associated with PHF-like hyperphosphorylation of Tau in the entorhinal cortex and lower synapse contacts and mossy fiber differentiation in the hippocampus [79]. Indeed, the cycle of PHF-like Tau and its degradation in the condition of “hypometabolism” has been proposed to be a physiological mechanism in regulating neuron plasticity [80]. Recently, it has been suggested that hyperphosphorylation of Tau in the AD might represent a specific compensatory adaptation, underscoring the interplay between energy expenditure and Tau protein modification [81].

A cell bargain with its residential mitochondria for proper energy production. As mitochondria generate ATP, they also generate byproducts of reactive oxygen species (ROS) such as superoxide, hydroxyl radical, and hydrogen peroxide [82]. Undoubtedly, aging is the primary cause of mitochondrial dysfunction and excessive oxidative stress, and both could explain why aging is the profound risk factor of neurodegenerative diseases [83], including AD and PD [84]. It has been reported that dysfunctional mitochondria are accumulated in axons in the early stage of AD and mouse models [85]. This cellular change is reminiscent of Wallerian neurodegeneration, where excessive axon mitochondria have also been characterized in the time window before distal axon degeneration (see below). In AD and PD, mitochondrial dysfunction could further block electron transport and produce excessive superoxide H_2_O_2_ and other free radicals, forming a vicious circle to damage macromolecules, including Tau [82, 86]. Tau dimers could form by oxidation of Cysteine residue in the microtubule binding motif repeat through intra- and inter-disulfide bonding at Cys291 and Cys322 residues [87–89]. Extending this line of research to examine the interplay between mitochondrial respiratory stress and post-translational modification of Tau may help to decipher the pathophysiological mechanism of tauopathy.

Another plausible pathogenic mechanism linking Tau and mitochondria appears to associate with DNA damage in nuclear and mitochondria. For post-mitotic neurons, although neurons usually would not replicate their nuclear DNA, mitochondrial DNA, however, is continuously replicated [90]. Because the mammalian mitochondria lack a comprehensive DNA repair system, DNA damage in mitochondrial genome is likely passed on to the newly synthesized DNA [91]. The accumulation of mitochondrial mutations over time would cause mitochondrial dysfunction and generate ROS [92, 93]. Moreover, because Tau-induced mitochondrial oxidative stress could potentially stimulate mtDNA to release into the cytosol, and the cytosolic mtDNA might prime the cytosol DNA sensor in neurons and stimulate the cytosolic anti-foreign DNA signaling, which could potentially induce innate immune response and neuroinflammation [94].

## Autophagy defect hinders tau processing and the links to tauopathy

Christian DeDuve and his colleagues discovered that cells could digest their cytoplasm by lysosomes in states of starvation or stress, and termed lysosomal degradation processes in general “autophagy” (auto-self; phagy-eating) in his seminal work around 1960 [95]. Neurons are complex regarding the specific structural organization and the nature of end-diving. Therefore, neuronal autophagy is perhaps the most challenge regulation for this highly elaborated sophisticated degradation, which may require some refinements to not only processing the targets to retain homeostasis in health but also to prevent the vulnerability of neurodegeneration in disease [96, 97]. In the past few years, anatomical studies have revealed the autophagic defects of human brains in AD and other tauopathy disorders [98]. One of the underlying mechanisms may associate with the “homing” connection defects due to the long distance transportation of autophagic vacuoles generated in the synapses that travel through axons or dendrites to lysosomes which primarily localize in the soma [99]. However, many studies are still needed to further dissect the physiological function of autophagy as a protective mechanism from pathogenesis, and uncomplete autophagic accumulation in tauopathy associated neurodegenerative disorders [95]. Here, we summarize current knowledge about autophagy in health and in tauopathy.

As the autophagy-lysosome pathway has several distinguishable marks, its links to tauopathies have been investigated. Emerging reports have indicated a massive accumulation of autophagic vacuoles and lysosomes in the brain sections from patients with Tauopathies, suggesting a defect of the autophagosome-lysosome pathway that contributes to Tau pathology. Massive autophagic defect rather than too much autophagy induction is associate with axonal and dendritic dystrophy where their compartment integrity was lost [100, 101]. Thus, it seems that autophagy induction is adequately regulated and activated when neurons need to combat Tauopathy or other proteinopathies, otherwise it would remain at the basal level. The big picture is whether autophagy stimulation ultimately is a protective mechanism for Tauopathy disorders and reduce Tau burden [102]? Autophagy is usually inhibited by mammalian target of rapamycin (mTOR), an evolutionary convergent cell-growth regulator that integrates growth factors, nutrient signals and cellular stress by upregulating protein synthesis. Pharmacological studies in human tauopathy animal models via the administrations of autophagy inducers (trehalose and methylthioninium chloride), or mTOR inhibitors (rapamycin, bexarotene, and galectin) support the notion that the involvement of autophagy in Tauopathy may be beneficial [103, 104]. Some studies demonstrated that Tauopathy appears to disrupt the axonal retrograde transportation of autophagosome by impairing the dynein-dynactin complex, but whether this physiological defect is a cause or the effect of Tau mischief remains to be clarified [105].

Understanding the temporal and spatial dynamics of autophagosome formation in the association with pathological Tau accumulation in the dystrophic neurons would be an essential investigation. Furthermore, as the functions of many Atg proteins have been discovered, perhaps the genetic and biochemical approaches on dissecting the link between the induction Atg genes and Tau modifications could address this intertwined problem with a straight answer [100].

Additionally, exosomes and exophers are two alternative pathways for the quality control in cells. It has been shown that Tau pathology could spread via the trans-synaptic connections and several potential mechanisms have been proposed, including unconventional protein secretion and exosome-mediated spreading. Several studies using biochemical and ultrastructure anatomical analyses supported that the inter-neuronal Tau propagation might be operated via exosomes from presynaptic neurons, which also hijack the endosomal pathway to penetrate receiving neurons [66, 106, 107]. This unconventional secretion of exosome-associated tau has been detected in human CSF samples for the AD patients [108]. As exosome-mediated exertion of unwanted/toxic proteins and materials might coordinate with autophagic regulation, it would be interesting to delineate their pathological roles in Tauopathies. Recently, work from *C. elegans* identified exophers could act as a large vacuole to removing the dysfunctional mitochondria and lysosomes from neurons expressing a neurotoxic, aggregable variant of huntingtin [109]. It will be interesting to investigate if a similar mechanism is also operated in removing various Tau aggregates that might be crucial for our understanding of the concept of trans-neuronal autophagy play such roles of tauopathy in brain tissues.

## Propagation of tau pathology in neurological diseases

Tauopathy in human is believed to last several decades in prodromal phase with NFT development with no significant clinical symptoms [110]. Axon pathology is the early neuropathology preceding the late phase of axon destruction in tauopathy-associated neurodegenerative diseases. In the tauopathy disease context such as AD, three distinct progressive stages have been proposed: the preclinical high-risk stage, mild cognitive impairment stage, and the disease manifestation stage [111]. While tauopathy is still a medical condition without a cure or a disease-modifying therapeutic strategy [112], some preclinical animal models have been established which aim to understand axon degeneration and to potentiate the study of axon regeneration and repair [113–117]. In the AD, a systematic pathological examination showed the accumulation of hyperphosphorylated Tau is initiated in axonal processes [81], and these aberrant Tau proteins subsequently fill in the somatodendritic compartment in affected neurons [118]. Detailed protein analysis found pathogenic Tau proteins assemble into oligomeric form and paired helical filament (PHF, pre-tangle stage) before eventually buildup neurofibrillary tangles (NFTs) in the degenerating neuronal cell bodies [119, 120]. Braak and colleagues analyzed many postmortem brains and concluded a stereotypic pattern of tau pathology where NFTs formation was first observed in the entorhinal cortex, followed in the hippocampus and some parts of the neocortex, and eventually spread out to the occipital lobes as well [121]. While the exact speed of Tau pathology propagation in different regions of the AD brain is not defined, it has been estimated that 50 years may be required from the pre-tangle stage to the full-blown stage of NFTs in AD brain [119]. Given such stereotypic pattern of tauopathy progression, it seems to suggest that intraneuronal NFTs in the entorhinal cortex may propagate the Tau aggregates through some types of cross synapse transmission to the post-synaptic neurons in the hippocampus. This slow-spreading of NTFs to the next connective targets thus may depend on the functional connections but not nearby vicinity [122, 123].

The axonal connectivity of neurons in a mature animal brain is mostly stable in macroscale. However, we do know that neurons can rewire their connections through pruning and regeneration [124]. How neurons maintain their axonal quality concerning regular physiological needs, and whether neurological disorders dictate the change of axon stability be one of the critical questions remain to be answered. In recent years, the concept of “prion-like” trans-synaptic spreading of Tau pathology has been proposed, hinting unrevealed mechanisms in regulating pathogenic Tau proteins to muddling functional synapses [125–127]. Some recent findings have endorsed the hypothesis of prion-like, trans-synaptic Tau spreading in AD patient brains and mouse models. Of note, others including whom support metabolic vulnerability [128] have dismissed the idea of Tau propagation crossing synapses, and debates are continuing.

## The distinct tau pathologies in various neurodegenerative disorders

The diagnose of different neurological disorders rely on patients’ functional disability with the assumption about that the distinct brain lesion could be explained to their anatomical manifestation from medical images (www.healthline.com/health/brain-disorders). Functional magnetic resonance imaging (MRI) (www.fmridc.org) and positron emission tomography (PET) in vivo brain imaging could detect functional connectivity imaging and Tauopathy using PHF tau ligands but the longitudinal analysis of changes across the brain associated with age and sex, is not yet common [129]. Until now, to distinguish the spectrum of Tauopathies in brain regions in regarding to different neurological disorders still primary depends on the examination of postmortem brains, although recent several clinical PET studies has attempted to correlate in vivo tau pathology with Braak stage definitions [121].

Historically, the neuropathological changes of Tauopathy in AD are referred to NFT which could be revealed by the silver staining [130, 131]. Nevertheless, detailed ultrastructural characterization of NFTs revealed specific Tauopathy entity that consists of tangled bundle of paired helical, straight Tau filaments, and twisted ribbon-like filaments (PHF, SF, and TRF respectively) [51, 131]. Each of these three Tau filaments can dominantly exhibit in a different spectrum of Tauopathy disorders. For example, PHF, with 8–20 nm in diameter and 80 nm in periodicity consists 95% of Tau filaments in AD, the rest of 5% is made by SF. A similar composition of Tau filaments is also reported in Down syndrome, Parkinsonism-dementia complex of Guam, and FTDP-17 [132]. On the other hand, SFs are the primary Tau filaments, and PHF-like but twisted filaments are the constituents of Tauopathy in Pick’s disease, progressive supranuclear palsy and argyrophilic grain disease. Furthermore, unique twisted ribbon-like Tau filament was observed in multiple system Tauopathy with presenile dementia (MSTD). Given the similarity of the ultrastructure between SF and PHF, it is predicted that the seeding of Tau might favor one form over the other [133]. It is important to know that Tau filaments could differ in their makeup by the ratio of six different Tau isoforms. For example, Tau filaments are composed in the 1:1 ratio of 3R and 4R tau in AD, but may not be the case in other Tauopathy conditions. In addition, unique ultrastructure features presented in each neurodegenerative disorders with “positive accumulation” of abnormal Tau filaments may differ in biochemical features of 3R or 4R or mixed Tau, and ins and outs of Tau propagation [134], which may associate with end phase of “negative loss” of axonal connections and synapses in various neurodegenerative conditions.

## Axonal degeneration in tauopathy and Wallerian degeneration

Tau is primarily associated with axonal microtubules, and it does not surprise the axonal pathology in white matters precedes the apparent cell death in AD brains. The appearance of tau aggregation within dystrophic neuritis, known as neuropil threads, is the early neuropathological makeup of tauopathy [86]. Therefore, understanding the manifestation of early phase tauopathy may be crucial for the design of rational therapeutic strategies for better intervention. Fortunately, the recent advance of AD neuroimaging initiative (www.adni-info.org) focusing on the axonal track trajectory has provided an opportunity to study the neurodegeneration in early stages of AD. Understanding the mechanism of similar conditions that could slow down axon degeneration may prove to be valuable for AD treatment.

More than 150 years ago, Waller observed that after severing the peripheral axons in frogs, the part of injured axon rapidly degenerate beginning via distal to proximal axon trajectory toward soma. This degenerative pattern is now named Wallerian degeneration [135]. Recent studies in this type of degeneration showed that degenerative process is not passive but a genetic program that can actively govern the subcellular self-destruction; and the Sarm1-MAPK pathway is likely to integrate the damage signal and instruct axon degeneration [136]. This Sarm1 genetic program is distinct from cell apoptosis because genetic deletion of apoptotic genes was unable to prevent axon destruction. Interestingly, degenerative phenotype could be delayed in a spontaneous mutant mouse, i.e. slow Wallerian degeneration (*Wld* ^S^). This *Wld* ^S^ gene encodes a chimera of Ube4b and nicotinamide mononucleotide adenylyltransferase (Nmnat1); and its expression can suppress axon destruction in neurodegenerative animal models and axotomy [137]. Axon self-destruction in the PNS could be followed by axon regeneration [138], but why most CNS neurons have difficulty in regenerating axons? Understanding how the environment and genetic modifications deprive such capability of axon regeneration in CNS may help us to identify targets for treating tauopathy-associated conditions which delay axon degeneration serves an early prevention.

## Conclusion

The most significant risk factor for AD and PD is age. It is a mysterious why Tau protein becomes mishehaving that let the aged neurons entering the paths to lure Tau become toxic seed and spread. The cruel and destructive tau seeds propagate and mishehaving breed with normal tau with puzzles of how they sweep through anatomic connectomicsh. Regarding Tau protein deposition, large-scale examinations of normal, pre-symptomatic, and diseased brains revealed that brain associated with tauopathy could develop over several decades, begins Braak I, and progresses to Braak VI stages [139, 140]. As Tau protein in the cerebrospinal fluid can be used as an early disease detection marker of tauopathy (www.alzforum.org/alzbiomarker) [112], the strategies to untangle tauopathy can be classified into two main groups: Tau-based or non-Tau-based. The central theme of tau pathology is a sequential protein transformation over time beginning with abnormal Tau hyperphosphorylation or post-translation modification to tau oligomer and neurofibrillary tangle formation. Therefore, the Tau-based strategy is to reduce or slow-down Tau pathology. On the other hand, the non-Tau based approach is centered around “time” to turn off the aging effect on the tauopathy progression by preserving brain homeostasis to mitigate aberrant Tau-induced damage.

## Tau-based strategy

As of 2017, there are nine Tau-based intervention agents in the pipeline of clinical trials (https://clinicaltrials.gov/ct2/home). These are all anti-Tau vaccines that aim to boost immunogenicity to prevent the seeding of tau aggregation and the potential spreading. The first immunotherapy strategy developed by Axon Neuroscience using AADvac1 vaccine is currently under phase III human trials. This anti-Tau vaccine comprises a synthetic peptide from amino acids 294 to 305 of tau (KDNIKHVPGGGS), which might prevent the residues 306–378 of tau proteins to adapt to beta/beta-helix structure of protofilaments [51]. While it is still early to say that this vaccine can rescue abnormal cognitive decline in the mild AD, some encouraging news reported the absence of pathologic Tau deposit in the wall of brain blood vessels.

## The non-tau strategy

Although AD and PD are considered as diseases prevalent in the elderly, the initiation of neuron pathology associated with tauopathy in the AD, PD, and other tauopathy-associated neurodegenerative diseases starts early before clinical symptoms are evident (www.alzinfo.org/.../brain-detected-20-years-alzheimers-symptoms) [141, 142]. During the progression of tauopathy, axonal transport defect, mitochondria dysfunction, and oxidative stress, all could impair neuronal function [143–146]. Therefore, time-based anti-aging and anti-oxidative damage mechanisms could be a useful strategy complementary to anti-Tau therapy [147]. As an example, Sinclair and colleagues suggested that one of the pathways that can decrease oxidative stress and DNA damage is by supplying the so-called “Golden Nucleotide” nicotinamide adenine dinucleotide (NAD^+^) [148]. NAD^+^ repletion may benefit DNA repair and decelerate neuron aging process [149, 150]. The treatment of nicotinamide is currently in clinical trial, and it appeared to reduce the phospho-Tau production from the CSF. Other strategies aim to enhance anti-oxidant and neurotransmission are also under clinical investigations.

## Abbreviations

AD: Alzheimer’s disease
EC: The entorhinal cortex
FTD: Frontal temporal dementia
FTDP-17: Frontotemporal dementia with parkinsonism-17
MAPT: Microtubule associated protein tau
mTOR: Mammalian target of rapamycin
NFT: **N**eurofibrillary tangles
PD: Parkinson’s disease
